# Mathematical Modeling of Vedolizumab Treatment’s Effect on Microbiota and Intestinal Permeability in Inflammatory Bowel Disease Patients

**DOI:** 10.3390/bioengineering11070710

**Published:** 2024-07-12

**Authors:** Antonio D’Ambrosio, Annamaria Altomare, Tamara Boscarino, Manuele Gori, Paola Balestrieri, Lorenza Putignani, Federica Del Chierico, Simone Carotti, Michele Cicala, Michele Pier Luca Guarino, Vincenzo Piemonte

**Affiliations:** 1 Unit of Chemical-Physics Fundamentals in Chemical Engineering, Department of Science and Technology for Sustainable Development and One Health, University Campus Bio-Medico of Rome, Via Alvaro del Portillo 21, 00128 Rome, Italy; tamara.boscarino@unicampus.it (T.B.); v.piemonte@unicampus.it (V.P.); 2 Department of Sciences and Technology of Sustainable Development and Human Health, Università Campus Biomedico di Roma, Via Alvaro del Portillo 21, 00128 Rome, Italy; a.altomare@policlinicocampus.it; 3 Gastroenterology Research Unit, Università Campus Bio-Medico di Roma, Via Alvaro del Portillo 21, 00128 Rome, Italy; manuele.gori@cnr.it (M.G.); m.cicala@policlinicocampus.it (M.C.); m.guarino@policlinicocampus.it (M.P.L.G.); 4 Institute of Biochemistry and Cell Biology (IBBC), National Research Council (CNR), International Campus “A. Buzzati-Traverso”, Via E. Ramarini 32, Monterotondo Scalo, 00015 Rome, Italy; 5 Gastroenterology Unit, Fondazione Policlinico Campus Bio-Medico di Roma, Via Alvaro del Portillo 200, 00128 Rome, Italy; p.balestrieri@policlinicocampus.it; 6 Units of Microbiomics and Human Microbiome, Bambino Gesù Children’s Hospital, IRCCS, Piazza Sant’Onofrio 4, 00165 Rome, Italy; lorenza.putignani@opbg.net; 7 Unit of Human Microbiome, Bambino Gesù Children’s Hospital, IRCCS, Piazza Sant’Onofrio 4, 00165 Rome, Italy; federica.delchierico@opbg.net; 8 Microscopic and Ultrastructural Anatomy Research Unit, Department of Medicine and Surgery, Università Campus Bio-Medico di Roma, 00128 Rome, Italy; s.carotti@policlinicocampus.it

**Keywords:** inflammatory bowel disease, vedolizumab, gut microbiota, intestinal permeability, compartmental model

## Abstract

Growing evidence suggests that impaired gut permeability and gut microbiota alterations are involved in the pathogenesis of Inflammatory Bowel Diseases (IBDs), which include Ulcerative Colitis (UC) and Crohn’s Disease (CD). Vedolizumab is an anti-α4β7 antibody approved for IBD treatment, used as the first treatment or second-line therapy when the first line results in inadequate effectiveness. The aim of this study is to develop a mathematical model capable of describing the pathophysiological mechanisms of Vedolizumab treatment in IBD patients. In particular, the relationship between drug concentration in the blood, colonic mucosal permeability and fecal microbiota composition was investigated and modeled to detect and predict trends in order to support and tailor Vedolizumab therapies. To pursue this aim, clinical data from a pilot study on a cluster of 11 IBD patients were analyzed. Enrolled patients underwent colonoscopy in three phases (before ($t_{0}$), after 24 weeks of ($t_{1})$ and after 52 weeks of ($t_{2\;}$) Vedolizumab treatment) to collect mucosal biopsies for transepithelial electrical resistance (TEER) evaluation (permeability to ions), intestinal permeability measurement and histological analysis. Moreover, fecal samples were collected for the intestinal microbiota analysis at the three time points. The collected data were compared to those of 11 healthy subjects at $t_{0}$, who underwent colonoscopy for screening surveillance, and used to implement a three-compartmental mathematical model (comprising central blood, peripheral blood and the intestine). The latter extends previous evidence from the literature, based on the regression of experimental data, to link drug concentration in the peripheral blood compartment with *Roseburia* abundance and intestinal permeability. The clinical data showed that Vedolizumab treatment leads to an increase in TEER and a reduction in intestinal permeability to a paracellular probe, improving tissue inflammation status. Microbiota analysis showed increasing values of *Roseburia*, albeit not statistically significant. This trend was adequately reproduced by the mathematical model, which offers a useful tool to describe the pathophysiological effects of Vedolizumab therapy on colonic mucosal permeability and fecal microbiota composition. The model’s satisfactory predictive capabilities and simplicity shed light on the relationship between the drug, the microbiota and permeability and allow for its straightforward extension to diverse therapeutic conditions.

## 1. Introduction

The intestine’s inner walls are lined with a functional unit organized as a multilayer system, called the gut barrier [1]. The latter spans approximately 300 m^2^ and consists of four layers, including the microbiota, mucin, the epithelial cell layer and Gut-Associated Lymphoid Tissue (GALT), which enable selective permeability, facilitating nutrient absorption and safeguarding against pathogens [2]. Maintaining the integrity of the gut barrier depends on the proper functioning of cell junctions between the epithelial cell layer, namely tight junctions (TJ) and adherent junctions (AJ), which regulate intestinal permeability [3,4,5,6]. The latter is, in turn, profoundly influenced by the gut microbiota, a collection of microorganisms that inhabit the digestive tract and produce vital enzymes required for nutrient assimilation, vitamin metabolism regulation and interaction with the immune system [7,8]. The microbiota’s composition varies throughout the gastrointestinal system, depending on genetic, nutritional and environmental factors [9]. It comprises over 50 species, with four primary phyla: *Firmicutes*, *Bacteroidetes*, *Actinobacteria* and *Proteobacteria* [10,11]. However, various factors, including psychophysical stress, diets that are high in fats and proteins but low in fiber and, particularly, specific pathologies, such as Inflammatory Bowel Disease (IBD), can lead to dysbiosis, an alteration in the gut microbiota that is associated with increased intestinal permeability to pathogenic agents [12,13].

Currently, it is estimated that more than 6.8 million people worldwide are affected by IBD, and this number is steadily increasing due to the Western lifestyle, urbanization and industrialization [14,15]. The main pathologies belonging to the IBD category are Crohn’s Disease (CD) and Ulcerative Colitis (UC), whose pathophysiological mechanisms are both characterized by uncontrolled activation of the enteric immune–inflammatory system [16,17]. More specifically, individuals with IBD exhibit a significant increase in dendritic cells and macrophages in the intestinal mucosa as a response to factors released by the altered microbiota, along with increased permeability [18]. This results in the release of a high quantity of pro-inflammatory cytokines, which activate both local and circulating lymphocytes and direct them to the site of inflammation. Lymphocyte migration within the intestinal lumen occurs via the binding of integrins, which are heterodimeric receptors placed on their surface, to adhesion molecules found on the surface of vascular endothelial cells [19].

It is well known that gut barrier dysfunction can be one of the main factors involved in the pathogenesis of IBD [19,20]. Specifically, mucosal addressing cell adhesion molecule-1 (MAdCAM-1), infiltrating CD4+ and α4β7 integrin t-cells have been associated with gut chronic inflammation in IBD [21].

Around 20% of patients do not respond to anti-TNF-α treatment, and the risk of loss of response is estimated to be up to 20% per patient/year [22,23]. This has led to the development of therapies utilizing specific anti-integrin monoclonal antibodies, which can impede the migration of T lymphocytes from the bloodstream to the enteric wall compartment, thereby preventing the recruitment of activated immune cells to inflamed intestinal tissues [19,20]. Vedolizumab, an innovative monoclonal antibody, belongs to this category. This drug can block lymphocyte trafficking to the intestinal epithelium by selectively binding to the α4β7 integrin present on the surface of lymphocytes [24]. This results in the inhibition of the interaction between T lymphocytes and MAdCAM-1, an adhesion protein expressed by intestinal endothelial cells that is upregulated in IBD patients [25]. 

Vedolizumab pharmacokinetics and pharmacodynamics have been extensively evaluated with the aim of analyzing the drug concentration over time and the effect on α4β7 integrin concentration and MAdCAM-1 expression [26,27,28,29,30,31,32]. Some pharmacokinetic parameters are listed in Table 1.

However, to the best of our knowledge, there are few, if any, physiologically based pharmacokinetic (PBPK) models in the literature that describe the pharmacokinetic properties of Vedolizumab. In general, the objective of a PBPK model is to describe the time-dependent distribution and disposition of a substance within a living system, utilizing principles derived from physiology, thermodynamics, anatomy and transport phenomena [33,34]. This can be achieved by representing living organisms as a series of lumped compartments that correspond to specific organs or tissues. Typically, these are arranged in a flow diagram and are characterized by a constant concentration of the targeted substance [35]. The applications of PBPK models are diverse and encompass a range of fields, including drug design and development [36,37] and clinical support [38,39,40]. For instance, these models can be employed to ascertain the most efficacious drug scheduling and dosage regimens, as well as to gain fundamental insights into the transport and metabolism of a substance in vivo [33]. Regarding Vedolizumab, the only complete PBPK model that has been developed is that proposed by Rosario et al. [41]. It comprises the central blood compartment, where the drug is injected, and the peripheral blood compartment, where Vedolizumab is consumed according to first-order kinetics. Moreover, the model incorporates a pharmacodynamics evaluation, which examines the relationship between Vedolizumab concentration in the peripheral blood compartment and the percentage of MAdCAM-1 binding by lymphocytes expressing high levels of α4β7 integrin. 

Recent findings demonstrated that Vedolizumab can significantly restore colonic epithelial permeability to ions in IBD patients, possibly predicting clinical responses [42]. Based on these data, the objective of the present study is to develop a mathematical model that simulates the effect of Vedolizumab in the gut. In particular, the model proposed by Rosario et al. [41] was extended in order to correlate the pharmacokinetics of Vedolizumab with its pathophysiological mechanisms of action. This approach permitted the delineation of a connection between the drug dosage and the observed variations in both intestinal permeability and fecal microbiota composition, offering a valuable tool for supporting and tailoring the treatment regimen.

## 2. Materials and Methods

### 2.1. Study Design

**Figure 1:**
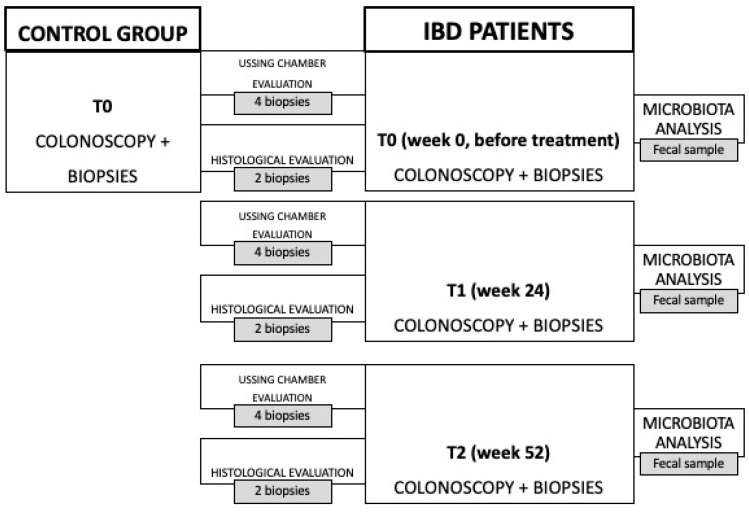
Flow chart of the pilot clinical study [42].

To assess the effects of Vedolizumab on IBD patients and to develop a mathematical model, a protocol was established as part of a recently published 24-month pilot clinical study [42], including enrollment and follow-up. Eleven patients diagnosed with moderately to severely active UC or CD were selected from outpatients and inpatients of the Fondazione Policlinico Universitario Campus Bio-Medico in Rome, including both individuals who had not undergone previous biological therapies (referred to as naïve) and those who had been formerly treated with anti-TNF medications. The patients were given a 300 mg IV dose of Vedolizumab (Entyvio 300 mg, Takeda Pharma A/S, Taastrup, Denmark and Takeda Manufacturing GmbH, Wien, Austria) during weeks 0, 2 and 6 (induction phase), and every 8 weeks thereafter (maintenance phase), until week 52. All IBD patients underwent endoscopy of the lower abdomen to define disease activity through the Harvey–Bradshaw Index (HBI) for CD patients [43] and the Partial Mayo Score (PMS) for UC ones [44], and to collect biopsies in the following three phases for transepithelial electrical resistance (TEER) evaluation, the measurement of intestinal permeability to a paracellular probe and histological analysis: before treatment ($t_{0}$), after 24 weeks of Vedolizumab therapy ($t_{1}$) and after one year of treatment ($t_{2}$). Moreover, fecal samples were collected at the three time points for intestinal microbiota analysis. In each phase, fecal microbiota analysis, TEER measurement, intestinal permeability evaluation and histological analysis were conducted. To ensure the quality of the results, a control group of 11 healthy gender- and age-matched volunteers was recruited and underwent colonoscopy and three sigmoid colon biopsies in order to evaluate the function and integrity of the mucosa. A schematic flow chart of the pilot clinical study is presented in Figure 1.

### 2.2. Data Analysis

To implement the mathematical model, data from 11 patients diagnosed with either CD or UC and treated with Vedolizumab according to the experimental protocol were used and compared to data from 11 healthy subjects (controls, CTRLs) [42]. They included both individuals who had not undergone previous pharmacological therapies (referred to as naïve) and those who had been formerly treated with anti-TNF medications. The sample considered is shown in Table 2.

Gut microbiota profiling was conducted via 16S rRNA region sequencing from the fecal samples of patients. fastq files, obtained from the Illumina sequencing platform, were analyzed using QIIME2 (v2023.2) [45]. The QIIME2 plugin for DADA2 was used for Amplicon Sequence Variant (ASV) table construction [46]. Taxonomic analysis was performed using a Naive Bayes model pre-trained on Greengenes2 2022 [47].

Mucosal barrier function was assessed using the gold standard method of Ussing Chambers, through which TEER was calculated [48]. Then, intestinal permeability was estimated on the basis of the paracellular passage of a 4kDa probe, fluorescein isothiocyanate-dextran (FITC-Dextran, hereafter referred to as FD4, 1 mg/mL), evaluated via the Ussing Chamber system [49]. Following this, intestinal permeability $P_{m}$ was estimated using the FD4 passage [50], according to Equation (1):

(1) $$P_{m}=\frac{Q_{t}\delta_{m}}{StC_{0}}$$

where $Q_{t}$ is the FD4 number of moles at time t, S is the area of the exchange surface of the intestinal membrane biopsy (equal to 0.017 cm^2^), $C_{0}$ is the initial FD4 concentration (namely 10^4^ pmol/cm^3^) and $\delta_{m}$ is the intestinal membrane’s thickness (equal to 2.175 × 10^−3^ cm). Finally, routine Hematoxylin and Eosin staining was performed in order to evaluate the inflammatory infiltrate, and the histopathological condition of mucosal damage was defined according to the Robarts Histopathological Index (RHI) at times $t_{0}$, $t_{1}$ and $t_{2}$ [51].

Overall, Excel spreadsheets and JASP software were used for statistical analysis of all data collected, reporting the mean, median, maximum, minimum and standard deviation. Additionally, various correlations between the data were investigated via paired samples *t*-tests and a Pearson Correlation Coefficient.

### 2.3. Mathematical Model

The objective of this study is to develop a mathematical model that can accurately depict the impact of Vedolizumab on intestinal permeability and microbiota composition. Thus, the initial step involves replicating the gastrointestinal physiology with respect to the concentration of the drug. 

**Figure 2:**
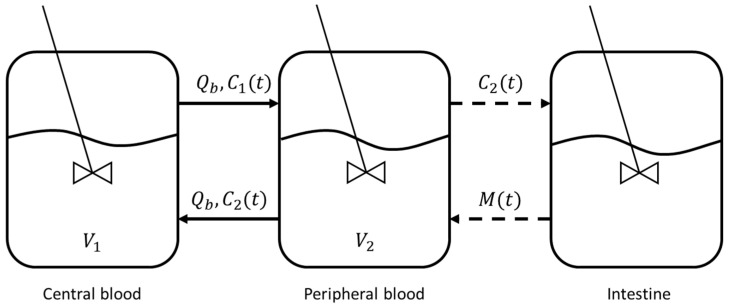
Representation of the three-compartmental model. Each compartment is described by a perfectly mixed reactor.

The three-compartmental model (comprising central blood, peripheral blood and the intestine) depicted in Figure 2, as described in Rosario et al. [41], was employed for this purpose, and the set of Equations (2)–(4) was used:

(2) $$\frac{dC_{1}(t)}{dt}=\frac{Q_{b}}{V_{1}}\left[C_{2}\left(t\right)-C_{1}\left(t\right)\right]$$

(3) $$\frac{dC_{2}(t)}{dt}=\frac{Q_{b}}{V_{2}}\left[C_{1}\left(t\right)-C_{2}\left(t\right)\right]-KC_{2}(t)$$

(4) $$M\left(t\right)-1=M_{0}\left[1-\frac{E_{max}{\left(C_{2}\left(t\right)\right)}^{\gamma }}{{\left(E_{50}\right)}^{\gamma }+{\left(C_{2}\left(t\right)\right)}^{\gamma }}\right]$$

where $C_{1}(t)$ and $C_{2}(t)$ are the concentrations of Vedolizumab in the central and peripheral blood compartments, respectively, while M(t) is the concentration of the endothelial adhesion molecule MAdCAM-1 in the intestine, which is directly influenced by drug concentration in the peripheral blood, according to Equation (4). The system of Equations (2)–(4) was solved through a MATLAB algorithm, based on the Euler finite difference method [52]. The initial conditions were $C_{1}\left(0\right)=60$ mg/L and $C_{2}\left(0\right)=0$, while the values of each parameter are listed in Table 3. Estimates were made from data from the literature [28,53] when values were missing.

Furthermore, the relationship between Vedolizumab concentration $C_{1}$ in the central blood compartment, the microbiota concentration $C_{b}$ (represented by *Roseburia* bacterial species) and intestinal permeability $P_{m}$ was investigated through experimental data regression, performed via the MATLAB Curve Fitter tool.

## 3. Results and Discussion

### 3.1. Fecal Microbiota Analysis

**Figure 3:**
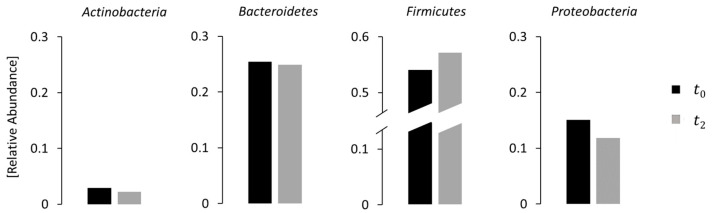
Comparison between microbiota compositions of IBD patients at phases $t_{0}$ and $t_{2}$ for the four major phyla (*Actinobacteria*, *Bacteroidetes*, *Proteobacteria* and *Firmicutes*). Data are expressed in relative abundance.

The results of the fecal microbiota analysis for IBD patients at phases $t_{0}$, $t_{1}$ and $t_{2}$ are visualized in Figure 3. The data related to the four main phyla (*Actinobacteria*, *Bacteroidetes*, *Proteobacteria* and *Firmicutes*) showed a decrease in *Actinobacteria*, *Bacteroidetes* and *Proteobacteria* and an increase in *Firmicutes* throughout the treatment, consistent with the existing literature [54]. 

**Figure 4:**
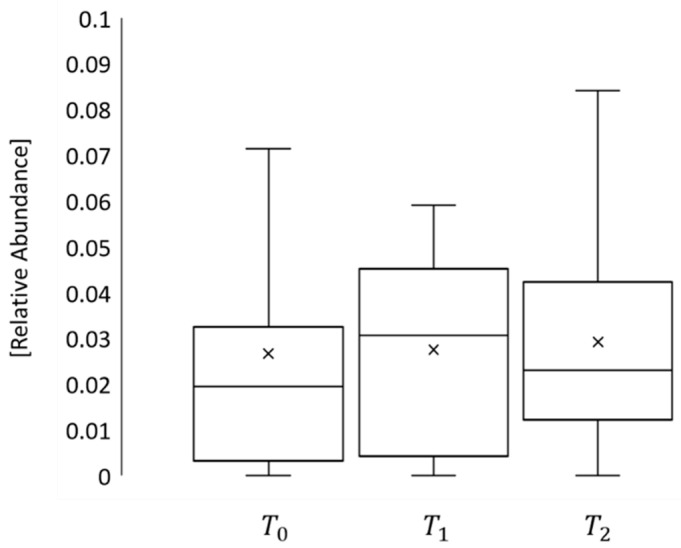
Bacterial density of *Roseburia* in phases $t_{0}$, $t_{1}$ and $t_{2}$.

Moreover, to explore the impact of Vedolizumab on microbiota composition, this study centered on *Roseburia*, a bacterial species belonging to the phylum of *Firmicutes*, whose abundance in IBD patients is typically diminished [55]. The results illustrated a rise in *Roseburia* levels from phase $t_{0}$ to $t_{2}$, as depicted in Figure 4, which is associated with an enhancement in intestinal anti-inflammatory function [56], arguably due to the medication’s influence.

### 3.2. TEER Measurement

**Figure 5:**
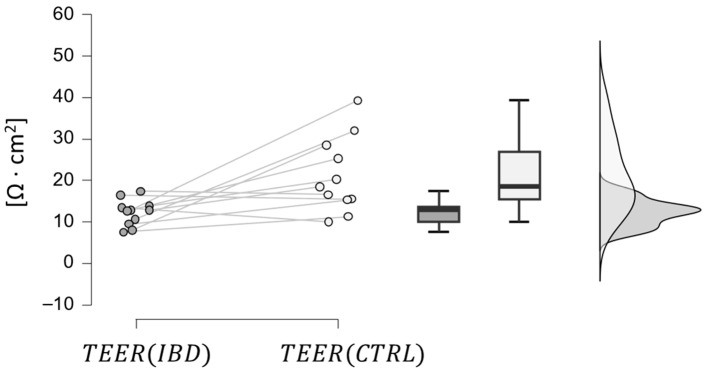
Comparison between TEER values in IBD patients at phase $t_{0}$ (dark grey) and in the control group.

**Figure 6:**
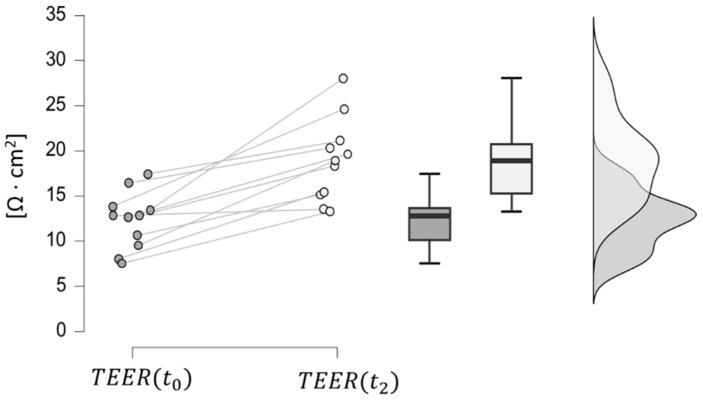
Comparison between TEER values at phases $t_{0}$ (dark grey) and $t_{2}$ (light grey) in IBD patients.

The results of the statistical analysis of the TEER measurement are reported in Table 4. Two main aspects are notable:

- IBD patients (at phase *t*_0_) have a lower TEER than healthy volunteers of the control group, which is coherent with IBD’s effect on intestinal epithelial TJs [57]. This hypothesis was confirmed by a paired samples *t*-test, which provided a *p*-value below 0.01, as depicted in Figure 5.
- In IBD patients, TEER increases from phase *t*_0_ to *t*_2_, reaching values similar to those of the control group. This trend indicates that the drug effect is restoring the function of the intestinal junctions, leading, in turn, to increased transepithelial electrical resistance to ion passage, since the paracellular resistance exerted by the TJ structure represents a major contributor to the TEER. This hypothesis was confirmed by a paired samples *t*-test, resulting in a significantly low *p*-value (less than 0.001), as shown in Figure 6.

### 3.3. Intestinal Permeability to Paracellular Passage Evaluation

**Figure 7:**
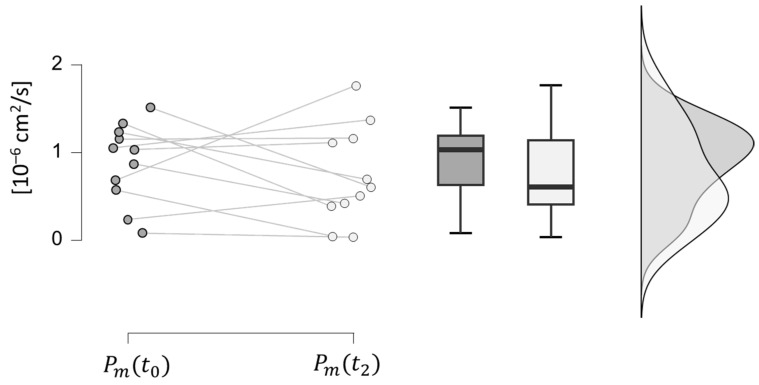
Comparison between intestinal permeability $P_{m}$ values at phases $t_{0}$ (dark grey) and $t_{2}$ (light grey) in IBD patients.

In Table 5, the statistical analysis results of the intestinal permeability to FD4 are presented. It is observed that IBD patients in phase $t_{0}$ have a higher value of permeability to FD4 than healthy volunteers, due to defects in the intestinal junctions caused by immuno-inflammatory mechanisms of chronic diseases [58] (this hypothesis was confirmed by a paired samples *t*-test with a significance level of 0.03). Additionally, it is noteworthy that the average intestinal permeability of FD4 among IBD patients declined from phase $t_{0}$ to $t_{2}$, in line with the rise in TEER values. However, when examining individual patients, a paired samples *t*-test determined that the hypothesis of increased intestinal permeability of FD4 at $t_{2}$ in comparison to $t_{0}$ cannot be disproven with a reasonable significance level (0.214), as depicted in Figure 7. Despite the therapy, Vedolizumab can only aid in restoring paracellular permeability values close to normal, as there remains a functional alteration of the intestinal barrier which cannot be fully recovered. This evidence aligns with the discovery of alterations in the genes responsible for maintaining mucosal integrity in IBD patients, resulting in a genetic predisposition to the pathology, which cannot be improved through therapies [59,60]. 

### 3.4. Histological Analysis

**Figure 8:**
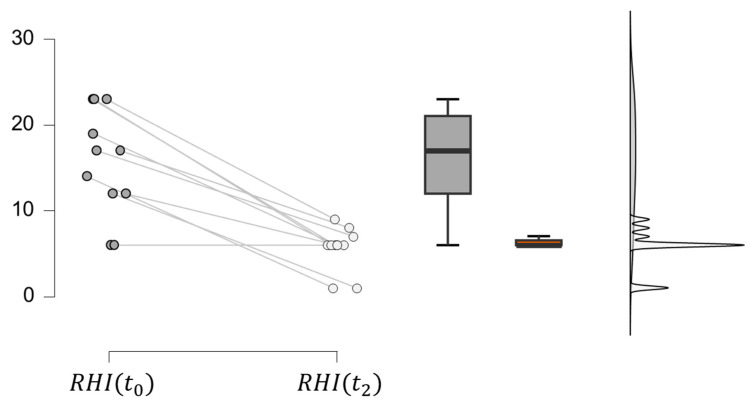
Comparison between RHI at phases $t_{0}$ (dark grey) and $t_{2}$ (light grey) in IBD patients.

The histological analysis led to the evaluation of the RHI for IBD patients, as reported in Table 6. A decrease in RHI is observed from the $t_{0}$ to the $t_{2}$ phase due to improved tissue inflammation as a result of Vedolizumab’s inhibitory effect on α4β7/MadCAM-1 binding, which prevents lymphocyte migration within intestinal tissue. This is confirmed by a paired samples *t*-test with a significance level below 0.001 (Figure 8). For many patients, moreover, there is no significant change in RHI from the $t_{1}$ to the $t_{2}$ phase. This observation is coherent with the drug’s mechanism of action, which produces 100% integrin saturation after the first administration. Indeed, it has been previously reported that Vedolizumab concentrations of around 1 μg/mL result in the complete saturation of α4β7 binding sites.

**Figure 9:**
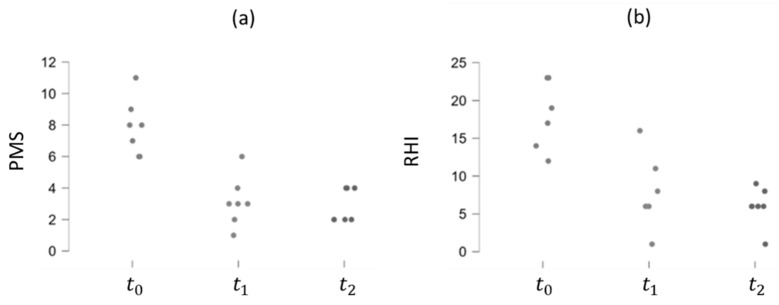
PMS (**a**) and RHI (**b**) trends over time in UC patients.

**Figure 10:**
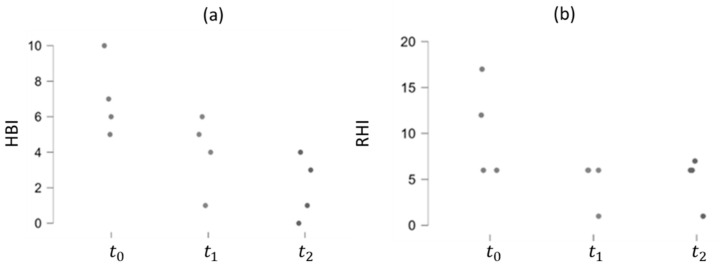
HBI (**a**) and RHI (**b**) trends over time in CD patients.

The correlation between the RHI values and the PMS and HBI indexes for UC and CD patients, respectively, was analyzed. Particularly, in UC patients, both RHI and PMS exhibit a decreasing trend over time, suggesting a positive effect of Vedolizumab therapy, as depicted in Figure 9. The Pearson Correlation Coefficient is, indeed, higher than zero (0.751), indicating a positive correlation between the two indexes (*p*-value < 0.01). This trend persists among CD patients, although the correlation between the RHI and HBI indexes is weaker (Pearson Correlation Coefficient = 0.651 and *p*-value = 0.011), as demonstrated in Figure 10.

### 3.5. Mathematical Modeling Results

**Figure 11:**
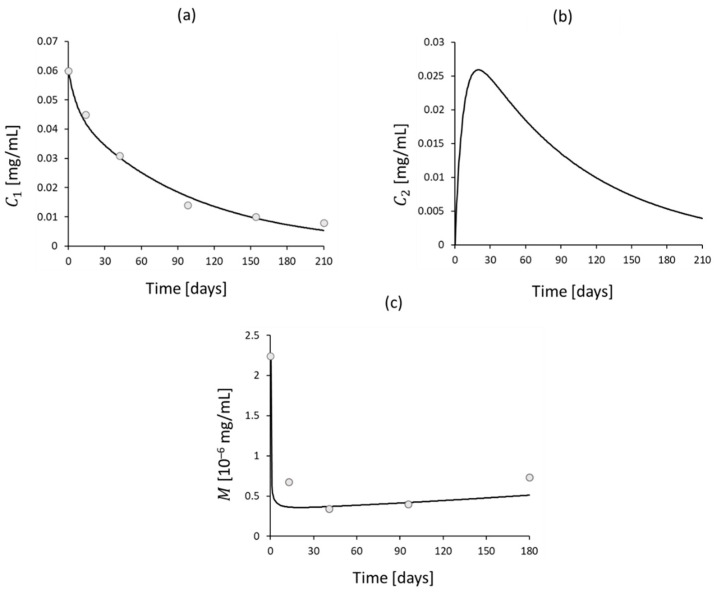
(**a**) Vedolizumab concentration in central blood compartment over time predicted by the model, compared to data from the literature [28] (dots). (**b**) Vedolizumab concentration in the peripheral blood compartment over time predicted by the model. (**c**) Intestinal concentration of MAdCAM-1 over time predicted by the model, compared to data from the literature [53] (dots).

The results of Equations (2)–(4) are depicted in Figure 11 and compared to the data from the literature [28,53], when available. The implemented mathematical model effectively represents Vedolizumab’s plasma concentration until week 14 of the maintenance phase. However, after this point, deviations are detected, with a faster decrease in the developed model. Regarding the concentration of the adhesion molecule MAdCAM-1, the model tracks the actual trend during the maintenance phase of the drug, while it inadequately represents the induction period.

**Figure 12:**
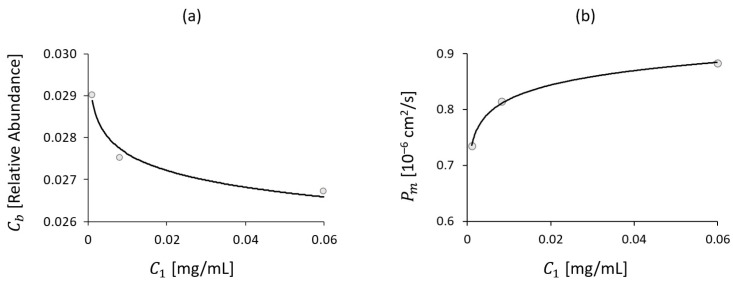
*Roseburia* abundance (**a**) and intestinal permeability (**b**) trends over Vedolizumab concentration in central blood compartment, as predicted by the model and derived from the clinical study (dots).

The trends of *Roseburia* abundance and intestinal permeability derived from the clinical study were compared with Vedolizumab concentration in the central blood compartment resulting from the model. Through experimental data regression, Equations (5) and (6) were obtained:

(5) $$C_{b}=-0.5750\cdot 10^{-3}\cdot \log C_{1}+2.4967\cdot 10^{-2}$$

(6) $$P_{m}=0.0370\cdot 10^{-6}\cdot \log C_{1}+0.9885\cdot 10^{-6}$$

where Vedolizumab concentration $C_{1}$ is expressed in mg/mL, *Roseburia* concentration $C_{b}$ in relative abundance and intestinal permeability $P_{m}$ in cm^2^/s. The logarithmic trends, depicted in Figure 12, adequately reproduce the clinical data ($r^{2}=0.9989$ for permeability and $r^{2}=0.9819$ for *Roseburia* abundance).

**Figure 13:**
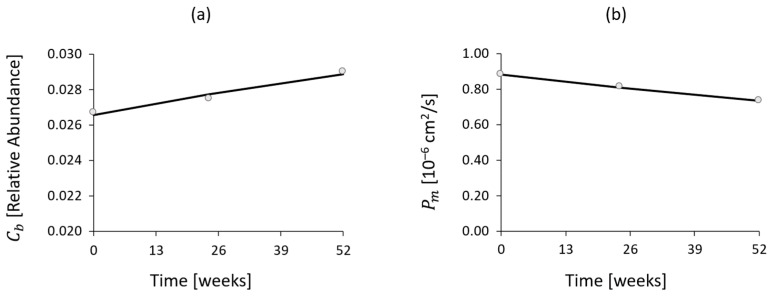
*Roseburia* abundance (**a**) and intestinal permeability (**b**) trends over time, as predicted by the model and derived from the clinical study (dots).

Furthermore, the mathematical model (Equations (2)–(4)) and fitting results (Equations (5) and (6)) were combined to derive the trends of *Roseburia* abundance and intestinal permeability as a function of time. This revealed a linear trend of increasing $C_{b}$ and decreasing $P_{m}$, which occurred in parallel with drug consumption (i.e., lowering $C_{1}$), as depicted in Figure 13. 

**Figure 14:**
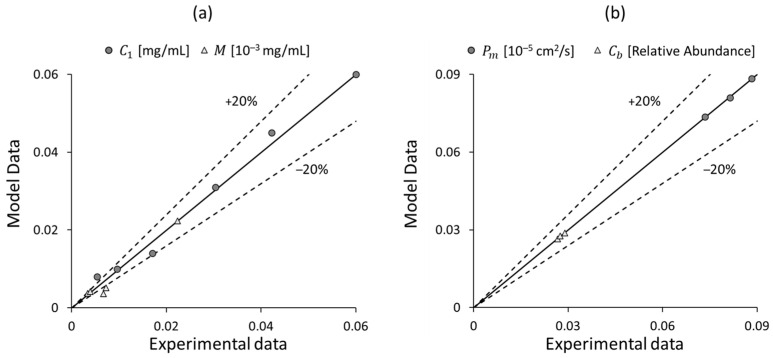
Parity plots between the model output and the experimental data for different variables: (**a**) the concentrations of Vedolizumab $C_{1}$ and MAdCAM-1 M, and (**b**) the intestinal permeability $P_{m}$ and *Roseburia* abundance $C_{b}$.

A summary of the results obtained is presented in Figure 14, which shows a parity plot between the model and experimental data.

The outcomes observed align with the findings derived from the pilot study [42], demonstrating that the model is capable of anticipating the physiopathological effects of Vedolizumab administration. Furthermore, the simplicity of the implemented equations allows for the ad libitum variation of certain parameters (e.g., the number of infusions and the initial dose) in order to assess the resulting change in intestinal permeability and *Roseburia* abundance. It is important to note that the model is subject to limitations related to the small number of patients, but it can be easily validated and extended for larger samples.

## 4. Conclusions

IBDs are complex diseases in which the physiopathological mechanisms are not completely understood. Several factors seem to be involved in response to new targeted therapy, such as the composition of the intestinal microbiota, intestinal permeability and inflammatory processes involving the intestinal mucosa and submucosa. The available data suggest that Vedolizumab can restore permeability values to normal levels. Furthermore, variation in microbiota composition was observed in patients who participated in the pilot study, with a trend of increasing *Roseburia* abundance, as shown in Figure 4. 

All the data obtained by the clinical study (Figure 5, Figure 6, Figure 7, Figure 8 and Figure 9) were incorporated into a three-compartmental mathematical model, which was developed in conjunction with data from the literature. This approach enabled the correlation of the concentration of Vedolizumab in the peripheral blood compartment with intestinal permeability and *Roseburia* abundance. On the whole, the model provides an adequate representation of clinical data, despite the inherent limitation of the reduced number of patients. It thus offers a simple and useful tool for anticipating the pathophysiological mechanisms of Vedolizumab therapies, supporting this treatment.

However, future research will include an expansion of this pilot study to increase the number of patients and to conduct further analyses. This will create a broader data set, thus enabling the validation of the preliminary results obtained. Moreover, the mathematical model can be refined and optimized by incorporating data on integrin and drug concentration in the blood and intestinal tissue of patients participating in the clinical trial. Ultimately, future research should delve into the relationship between dysbiosis and the upregulation of MAdCAM-1, considering the diverse phyla present in the gut, in order to facilitate the development of tailored therapies.

## Figures and Tables

**Table 1:** Vedolizumab pharmacokinetic parameters after a single IV dose [29,30].

	Vedolizumab Dose [mg/kg]			
	Healthy Volunteers		UC Patients	
Parameter	2.0 (n=7)	10.0 (n=7)	2.0 (n=10)	10.0 (n=11)
$C_{max}$ [μg/mL]	58.4	243	60.4	291.9
AUC [μg·day/mL]	955	4840	848	4373
$t_{1/2}\;[\mathrm{day}]$	14.1	14.8	15.1	20.6
CL [L/day]	0.164	0.139	-	-
$V_{z}$ [L]	3.28	2.73	-	-

$C_{max}$, maximum observed serum concentration; AUC, area under the drug concentration–time curve (evaluated until the time of the last quantifiable concentration for healthy volunteers and between days 0 and 14 and days 85 and 99 for UC patients); $t_{1/2}$, terminal estimation half-life; CL, total clearance; $V_{z}$, volume of distribution during the terminal phase.

**Table 2:** Patient sample considered for data analysis and relative progression of IBD pathology.

Patient Number	IBD	Therapy	HBI ($t_{0}$$\text{-}t_{1}$$\text{-}t_{2}$)	PMS ($t_{0}$$\text{-}t_{1}$$\text{-}t_{2}$)
1	UC	Naïve		8-4-4
2	UC	Naïve		8-2-2
3	CD	Naïve	6-6-4	
4	UC	Anti-TNF		6-3-4
5	CD	Naïve	7-5-1	
6	CD	Anti-TNF	5-4-3	
7	UC	Anti-TNF		11-1-2
8	CD	Naïve	10-1-0	
9	UC	Anti-TNF		9-3-2
10	UC	Naïve		7-3-2
11	UC	Anti-TNF		6-6-4

**Table 3:** Parameters adopted in the three-compartmental model.

Parameter	Notation	Value	Reference
Blood flow rate [L/g]	$Q_{b}$	0.12	[41]
Vedolizumab kinetic constant [g^−1^]	K	0.0368	[This Work]
Central blood compartment volume [L]	$V_{1}$	3.12	[41]
Peripheral blood compartment volume [L]	$V_{2}$	1.65	[41]
MAdCAM-1 initial concentration [mg/L]	$M_{0}$	2.23 × 10^−2^	[53]
Maximum Vedolizumab effect [⋅]	$E_{max}$	0.956	[41]
Hill coefficient [⋅]	γ	0.3512	[This Work]
Vedolizumab concentration at half-maximum effect [mg/L]	$E_{50}$	0.093	[41]

**Table 4:** Results of statistical analysis of TEER measurement for both IBD patients (at phases $t_{0}$, $t_{1}$ and $t_{2}$) and control group volunteers.

Parameter [Ω·cm^2^]	IBD Patients ($t_{0}$)	IBD Patients ($t_{1}$)	IBD Patients ($t_{2}$)	Control Group
Mean	12.27	18.12	18.36	21.15
Median	12.80	16.80	18.30	18.50
Std	3.15	7.23	4.86	9.14
Minimum	7.50	10.00	13.25	10.00
Maximum	17.40	34.50	28.00	39.32

**Table 5:** Results of statistical analysis of the intestinal permeability of FD4 for both IBD patients (at phases $t_{0}$, $t_{1}$ and $t_{2}$) and control group volunteers.

Parameter [10^−6^·cm^2^/s]	IBD Patients ($t_{0}$)	IBD Patients ($t_{1}$)	IBD Patients ($t_{2}$)	Control Group
Mean	0.883	0.814	0.735	0.686
Median	1.030	0.974	0.605	0.528
Std	0.451	0.446	0.552	0.365
Minimum	0.080	0.054	0.032	0.249
Maximum	1.510	1.450	1.760	1.270

**Table 6:** Histological analysis results for IBD patients at phases $t_{0}$, $t_{1}$ and $t_{2}$. RHI is not reported for the control group as it was zero for all healthy subjects (no tissue inflammation was detected).

Patient Number	RHI (*t*_0_)	RHI (*t*_1_)	RHI (*t*_2_)
1	17	8	8
2	12	6	6
3	6	6	6
4	14	1	1
5	12	1	1
6	6	6	6
7	23	6	6
8	17	6	7
9	23	11	6
10	23	16	9
11	19	6	6
Mean	16	7	6

## Data Availability

The data presented in this study are available on request from the corresponding author.
